# The Beat

**Published:** 2010-08

**Authors:** Erin E. Dooley

## FDA Urges Judicious Use of Antimicrobials in Livestock

In June 2010 the U.S. FDA issued draft guidance calling on food animal producers to use medically important antibiotics for food-producing animals only when necessary and with veterinary oversight.^1^ The agency proposes to phase in voluntary measures that would limit antimicrobial use in animals in a bid to limit the development of drug-resistant bacteria. The FDA is most concerned about limiting the use of drugs given to promote growth in animals and those that are administered continuously through feed and water. The draft guidelines will be open for comment through the end of August.

**Figure:**
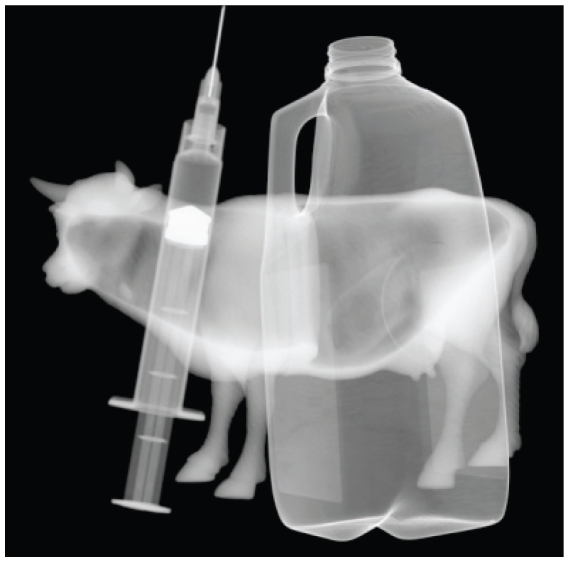


## Link Between Air Pollution, Temperature, and Sleep-Disordered Breathing

Researchers have found novel evidence for a link between air pollution and diminished sleep quality, a potential intermediate step toward cardiovascular disease.^2^ Using data from the Sleep Heart Health Study, the researchers found evidence that increases in PM_10_ and temperature independently affected nighttime hypoxia and sleep-disordered breathing, a group of conditions that includes sleep apnea and may affect up to 17% of U.S. adults. Although sleep-disordered breathing and air pollution have both been linked separately to an increased risk for cardiovascular disease, it is not yet known whether or how air pollution might adversely affect cardiovascular risk by increasing sleep-disordered breathing.

## Some Organic Pesticides Not So Clean

A two-year study has found that, compared with several new synthetic insecticides, some organic insecticides were more harmful to predator organisms (which help control target pests) and had a more negative overall environmental impact.^3^ In addition, in order to effectively control pests, organic pesticides often were used in higher volumes. The authors conclude that all pesticides must be evaluated using an empirically based risk assessment, “because generalizations based on chemical origin do not hold true in all cases.”

## Gulf Oil Spill Response Map

Geoplatform.gov/gulfresponse is a new online resource developed by NOAA in partnership with other agencies and stakeholders to offer near real-time data on the federal response to the *Deepwater Horizon* oil spill in the Gulf of Mexico. Visitors can use an interactive map to plot the latest available information about the spill’s trajectory, fishery closures, wildlife data, and locations of deployed research vessels. The map also highlights coastal areas where oil and tar balls have been observed and gives details about the extent of these problems and the environmental sensitivity classification of the affected areas.

**Figure:**
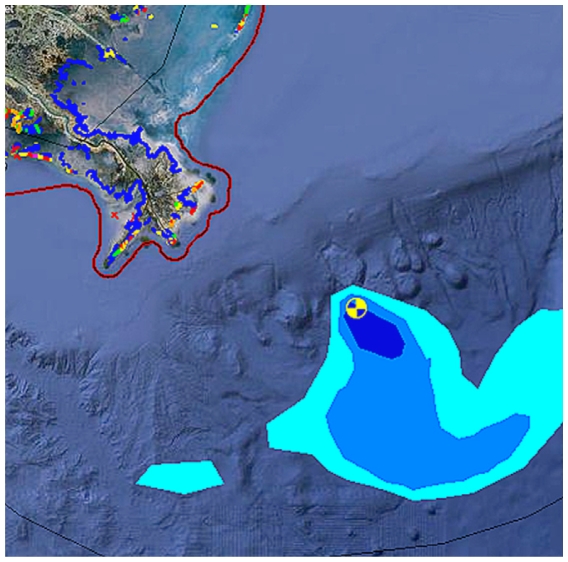
NOAA’s spill response map can be customized to show any combination of dozens of parameters.

## EPA Proposes New Power Plant Pollution Regs

Emissions from power plants can be transported hundreds of miles, affecting the health of populations far from the pollution’s source. The U.S. EPA has proposed regulations to curb emissions of sulfur dioxide and nitrogen oxides at their source.^4^ The proposed regulations would take the place of the 2005 Clean Air Interstate Rule, which the DC Circuit Court ordered the EPA to revise in 2008. The proposed regulations outline three possible approaches for emissions reductions, all of which involve some version of a cap-and-trade system.

## Oil Spills May Affect Seawater Arsenic Levels

Recently published work suggests oil pollution may render the seafloor unable to filter out arsenic that occurs naturally in the ocean and is introduced by drilling operations and oil spills.^5^ Sediments on the seafloor naturally bind arsenic, removing it from seawater. The authors of the new laboratory study found that low pH levels in seawater created a positive charge on samples of goethite (an iron oxide that is one of the most abundant compounds in ocean sediments), which then attracted negatively charged arsenic. Adding oil to the water created a physical barrier on the goethite and weakened the attraction between the two minerals. If oil pollution causes similar effects in ocean waters, the authors speculate arsenic may concentrate in the food chain to potentially harmful levels.
